# Minimally Invasive Approaches for Traumatic Rupture of the Pancreas in Children—A Case Series

**DOI:** 10.3390/children9081102

**Published:** 2022-07-22

**Authors:** Hannah Noemi Stundner-Ladenhauf, Leopold Bauer, Christian Heil, Josef Holzinger, Ottokar Stundner, Roman Metzger

**Affiliations:** 1 Department of Pediatric and Adolescent Surgery, Paracelsus Medical University Hospital, 5020 Salzburg, Austria; le.bauer@salk.at (L.B.); c.heil@salk.at (C.H.); r.metzger@salk.at (R.M.); 2 Department of Visceral, Transplant, and Thoracic Surgery, Medical University Innsbruck, 6020 Innsbruck, Austria; 3 Department of Surgery, Paracelsus Medical University Hospital, 5020 Salzburg, Austria; j.holzinger@salk.at; 4 Department of Anesthesiology and Intensive Care, Medical University Innsbruck, 6020 Innsbruck, Austria; ottokar.stundner@tirol-kliniken.at

**Keywords:** pancreatic injury, children, pediatric, abdominal trauma, organ laceration

## Abstract

Pancreatic trauma in children is rare; therefore, both scientific knowledge and clinical experience regarding its management are limited. Abdominal sonography and subsequent computed tomography (CT) imaging are the diagnostic mainstay after severe abdominal trauma in many pediatric trauma centers. However, the diagnosis of pancreatic injury is missed on the initial imaging in approximately one third of cases, with even higher numbers in young children. While conservative treatment is preferred in low-grade injuries, surgical interventions may be indicated in more severe injuries. We present a case series including four patients with high-grade pancreatic injury. Two patients were treated surgically with open laparotomy and primary suture of the head of the pancreas and pancreatico-enterostomy, one patient underwent endoscopic stenting of the pancreatic duct and one received conservative management including observation and secondary endoscopic treatment. We want to emphasize the fact that using a minimally invasive approach can be a feasible option in high-grade pancreatic injury in selected cases. Therefore, we advocate the necessity of fully staffed and equipped high-level pediatric trauma centers.

## 1. Introduction

Pancreatic trauma in children is rare, occurring in 3–12% of patients with abdominal injuries [1]. Due to these low numbers, both clinical experience and scientific knowledge regarding management of pancreatic trauma is very limited, especially when the pancreatic duct is involved [2]. Only few case reports and small case series contribute to the current knowledge [3,4,5]. Frequently, pancreatic injuries result from a motor vehicle or bike accident with blunt trauma to the abdomen [5]. Although pancreatic injury is uncommon overall, it represents the fourth most common abdominal organ injury, following spleen, hepatic and renal trauma [6]. Nonoperative management has become the standard of care for pediatric solid organ injuries other than pancreatic injuries, with success rates exceeding 90% [3]. However, the optimal treatment mode of pancreatic injuries—conservative, minimally invasive, or open surgical—is not clarified yet. Traditionally, open surgery was deemed obligatory for high-grade pancreatic injury. On the one hand, advocates of operative treatment argue that open surgery is associated with a faster recovery and significantly lower risk of pseudocyst formation [1,7,8,9,10]. On the other hand, minimally invasive management is emerging as a feasible alternative to open surgery even in high-grade cases. We present a case series including four patients with high-grade pancreatic injury, aiming to highlight differential approaches to the management of pancreatic injury.

## 2. Material and Methods

Demographic and clinical data on four consecutive patients with severe pancreatic injury were extracted from the hospital patient data information system of a level one pediatric trauma center over an 8-year period. Data analyzed included age, gender, mechanism of injury, mode of treatment (open surgical approach versus minimally invasive approach and conservative management), periprocedural care and long-term outcome. Data were fully and automatically anonymized before extraction from the clinical information system; extraction was performed by a third-party database operator using clinical diagnosis codes and age cutoffs. The study was exempted from ethical review by our institution’s clinical directorate.

## 3. Case Series and Results

**Figure 1:**
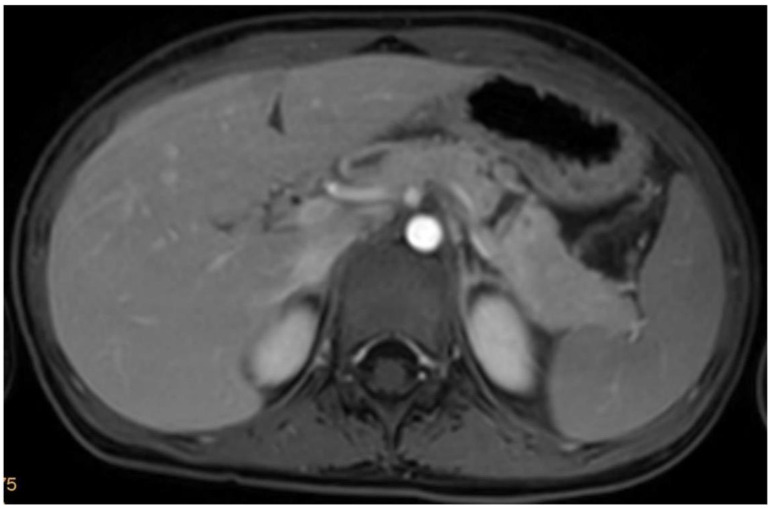
Complete rupture in the middle of the pancreas.

From 2010 to 2018, four patients—aged 4 to 13 years—with blunt abdominal trauma resulting in high-grade pancreatic injury were admitted to our institution. Two of those presented with an AAST grade III injury (Figure 1), whereas two presented with an AAST grade IV injury. Two were treated non-operatively, one underwent primary open surgical repair, and one was referred for surgical management after advanced diagnostic ERCP and concomitant jejunal perforation (see Table 1 for patient demographics, diagnosis, concomitant injuries, and timeline of treatment).

*Patient 1* was a 13-year-old male, suffering blunt abdominal trauma during a motor vehicle accident. The initial diagnosis was obtained from abdominal sonography and subsequent CT scans, revealing an AAST grade IV injury with complete proximal transection of the pancreas including the pancreatic duct. Due to the extensive injury to the pancreas, the decision was made to opt for open surgical management. Upon dissection of the pancreas, the pancreatic head and corpus were completely transected with disruption of the pancreatic duct; however, the ampulla Vateri was intact. The pancreatic head was sutured, and a Y-Roux anastomosis to reconnect the pancreatic body and tail performed. Postoperatively, the patient presented with a portal vein thrombosis with partial obstruction, which was successfully treated conservatively with anticoagulants.

*Patient 2* was a 7-year-old male who was referred to our center on post-accident day one with diagnosis of an incomplete rupture of the pancreas. Due to inconclusive CT imaging, ERCP with possible intervention was scheduled. However, on ERCP, complete dissection of pancreatic head and body (AAST IV) including complete rupture of the pancreatic duct was visible. Minimally invasive stenting of the pancreatic duct was unsuccessful; therefore, open surgical management was necessary. During open surgery, a rupture of the jejunum with a diameter of one centimeter was encountered and surgically sutured. Due to complete transection of the pancreas and pancreatic duct without option of surgical reconstruction, surgical management included suture of the pancreatic head and primary gastro-pancreatic anastomosis. However, it must be pointed out that internal drainage after distal pancreatectomy is only feasible in physiologically stable patients. Postoperative course of treatment was uneventful.

**Figure 2:**
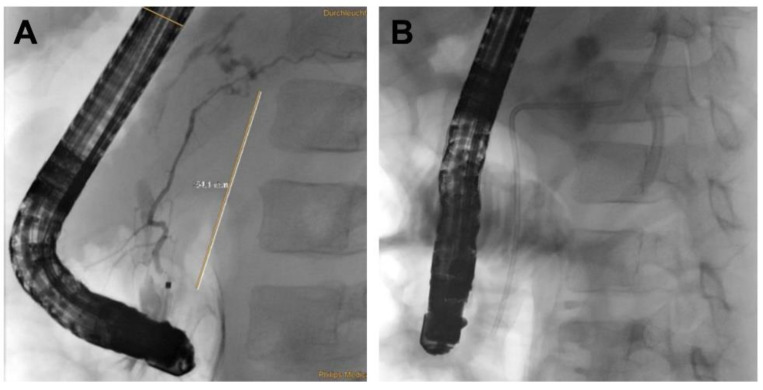
ERCP demonstrating the rupture of the pancreatic duct with paravasation of contrast dye (**A**). Stent placement (**B**).

**Figure 3:**
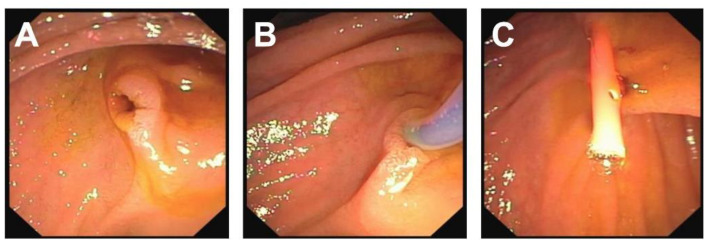
Major duodenal papilla leaking bile (**A**). Application of contrast dye into the principal pancreatic duct (**B**). Stent with multiple perforation in place (**C**).

**Figure 4:**
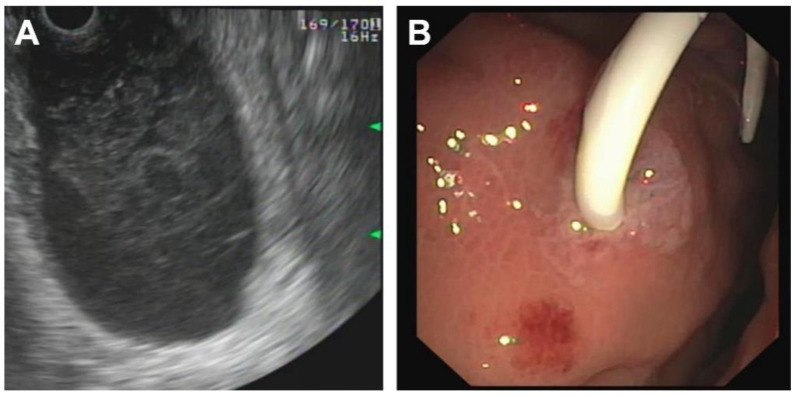
Transgastric endosonography showing a retention at the site of rupture (**A**). Transgastric placement of a pigtail drain (**B**).

**Figure 5:**
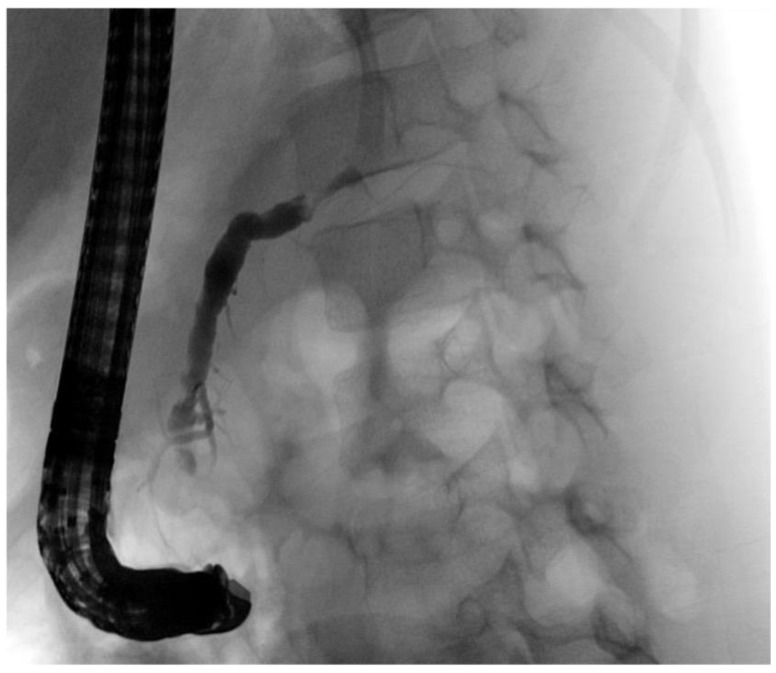
Follow-up ERCP after almost 6 months showing an intact principal pancreatic duct.

*Patient 3* was a 7-year-old female who initially presented nearly asymptomatic following a non-motorized scooter accident. Initial work-up included laboratory exams and abdominal sonography. Due to increasing pancreatic enzymes, minimal free fluid on sonography and recurrent vomiting, an MRI of the pancreas was obtained revealing AAST grade III injury to the pancreas including the pancreatic duct. Minimally invasive treatment was chosen, and the pancreatic duct was stented with a Ch 5 pigtail drainage during ERCP (Figure 2 and Figure 3). Postinterventionally, formation of a pseudocyst was encountered, which was drained with a double-pig-tail drain Ch 7 via a transgastric punction (Figure 4). Due to spontaneous dislocation of the pancreatic stent and subsequent infection of the pseudocyst with *Clostridium difficile*, antibiotic therapy and recurrent ERCP-stenting as well as transgastric drainage had to be performed (2 times). Overall, 5 months after initial intervention, the pancreatic stent was successfully removed (Figure 5).

*Patient 4* was a 4-year-old male, suffering from an AAST grade III pancreatic injury involving the pancreatic duct. For further diagnostic imaging and possible minimally invasive management, the patient was referred to ERCP. However, stenting of pancreatic duct was unsuccessful. Therefore, conservative management was initiated. During follow-up, formation of a pancreatic pseudocyst on post-accident day 10 with concomitant *Candida albicans* superinfection was diagnosed, which was treated with prolonged antifungal medication and single-time transgastric punction and subsequent drainage using a double-pig-tail Ch 7, which spontaneously dislocated. Further follow-up was uneventful.

## 4. Discussion

Obtaining a correct diagnosis after blunt abdominal trauma can be a great challenge. Recent studies have shown a substantially increased complication rate and long-term morbidity in patients with a lesion of the pancreatic duct; therefore, correct initial diagnosis and thorough assessment of the pancreatic duct are essential in every pediatric abdominal trauma [1,12].

In suspicion of severe abdominal trauma, most pediatric trauma centers rule out intra-abdominal fluid collection or organ lacerations using abdominal sonography, followed by computed tomography (CT) imaging [13,14]. Laboratory tests (liver and pancreatic enzymes) may add further information. However, in up to 40%, diagnosis of pancreatic injury is missed on abdominal CT studies in the first 12 h. Furthermore, sensitivity for ductal injury ranges around 50–55% on initial scans [15].

In case of inconclusive or dissonant findings between abdominal sonography and CT scans, endoscopic retrograde cholangiopancreatography (ERCP) and/or magnetic resonance cholangiopancreatography (MRCP) were shown to be the most reliable diagnostic modalities for pancreatic duct evaluation [16,17]. Additionally, ERCP comes with the benefit of providing an immediate treatment option in case of pancreatic duct injuries [12,18,19]. MRCP has become a viable, non-invasive alternative to ERCP for pancreatic duct imaging. Using MRCP, thorough investigation of the pancreatic duct, its duct upstream and a possible laceration as well as any parenchymal damage or peripancreatic fluid collections can be assessed. However, both modalities are time-consuming, resource intensive and often limited to specialized, tertiary-care hospitals [20,21,22]. Therefore, ERCP may not be available in all trauma centers; moreover, its performance in smaller children is technically challenging [18]. Therefore, referral of children with blunt abdominal trauma and suspected organ laceration to an experienced trauma center with the availability of pediatric ERCP must be recommended. However, given the right indication and availability of appropriately trained staff and equipment, minimally invasive treatment of pediatric pancreatic injury using endoscopic stenting may be a useful, safe, and less invasive alternative to conservative management or the traditional, open surgical approach.

Pancreatic injury can be divided into five categories in accordance with the American Association for the Surgery of Trauma (Table 2). Other modes of classification for pancreatic injury have been suggested in the literature, including the Takishima, Cape Town, and Lucas classifications [23].

While non-operative—conservative—management is preferred in low-grade (grade I and II) injuries, the best course of treatment to manage grade III, IV and V injuries remains controversial [25,26]. However, there also have been recommendations to aim for non-operative, non-internventional management in high-grade injury. In a retrospective cohort study including 11 children with a grade III or higher pancreatic injury by Goldberg-Murow et al., non-operative management was associated with a similar length of hospital stay when compared to operative management. However, Goldberg-Murow et al. [27], as well as several other studies, have shown that non-operative management of high grade injuries is associated with a higher rate of complications, such as pseudocyst formation, fistulas and in some studies an increased length of hospital stay [7,9,27]. A systematic review by Koh et al. showed pseudocyst formation in 18% of patients after non-operative management, but only 4% after operative treatment, suggesting that especially patients with additional injuries may benefit from a minimally invasive approach [28,29,30]. Wood et al. showed that there was no difference in the median length of stay between non-operative and operative management of grade III and IV patients [10].

ERCP is not only an effective diagnostic tool for evaluation of ductal integrity in pancreatic injury, but also affords the possibility of immediate therapeutic interventions. A minimally invasive approach can avoid laparotomy with all associated short- and long-term complications, including risk of hemorrhage, bowel and solid organ laceration, wound dehiscence, infection, prolonged pain, immobility, and intensive care admission [2,14]. There have been some concerns about ERCP in children, including technical difficulty of cannulating the smaller ampulla, increased risk of perforation, the occurrence of post-ERCP pancreatitis or peripancreatic leakage of pancreatic fluid as well as the need for repeated general anesthesia in younger children [18].

However, in the last decade, more experience has been gained using ERCP in very young patients. Houben et al. reported on 15 cases of pediatric pancreatic injuries from 1999–2004, 12 of which underwent ERCP, and nine stents were placed due to ductal injury or development of symptomatic fluid collection [19]. In total, 4 required a second endoscopy to exchange the stent to a larger caliber. ERCP was tolerated well, and only minor complications were noted, including transient rise in serum amylase (*n* = 5) and an exacerbation in epigastric pain for up to 48 h (*n* = 2). Management was successful, though median length of total parenteral nutrition and hospital stay were long (28 and 41 days, respectively).

In a retrospective study, Rosenfeld et al. reviewed 28 children with pancreatic injury who underwent ERCP [17]. In total, 3 patients underwent ERCP following operative management; the remainder had ERCP as an adjunct to non-operative management or underwent operative management following ERCP. In total, 15 patients received early ERCP within the first seven days. Overall, 4 of them were performed solely for diagnostic purposes, when duct integrity was unclear on CT scan. Duct perforation was detected in 2 patients, prompting operative management; 2 patients were downgraded from III to II, which avoided surgical intervention [17]. The other 11 patients underwent ERCP with primary therapeutic intent to attempt control of duct leakage by stent placement or sphincterotomy.

## 5. Conclusions

Data from this case series add to the currently limited knowledge on pancreatic injuries in children. In case of inconclusive imaging, ERCP can be an essential tool to evaluate suspected pancreatic injury and assess the pancreatic duct. Favorable outcomes in both patients with pancreatic duct injury who were treated with a minimally invasive approach suggest that ERCP can be a safe and feasible alternative to conservative or an open surgical approach in selected cases. However, our cases clearly demonstrate challenges and potential serious complications following ERCP. Therefore, attempting this treatment should be reserved for centers capable of appropriate complication management. However, since incidence rates of pancreatic injuries in children are very low, the generation of highly discriminatory data will remain a challenge—multi-center registry studies might be capable of generating such conclusions.

## Figures and Tables

**Table 1:** Patient characteristics and treatment timelines [11].

	Patient 1	Patient 2	Patient 3	Patient 4
Age	13	6	7	4
Sex	Male	Male	Female	Male
Cause of trauma	Bike accident	Motor vehicle accident (patient run over by agricultural vehicle)	Non-motorized scooter accident	Motor vehicle accident (collision as car passenger)
Initial diagnosis obtained by	Computed Tomography	Computed Tomography	Magnetic Resonance Tomography	Computed Tomography
Additional injuries	Retroperitoneal Hematoma	Jejunal Perforation, Lung Contusions, Unstable Pelvic Fracture (External Fixation)	None	Lung Contusions, Hepatic rupture, left pneumothorax
Grade of Pancreatic Injury	IV	IV	III	III
Initial Management	Laparotomy, suture of the pancreatic head, distal pancreato-jejunostomy (Roux Y)	ERCP—complete dissection of the pancreas, Laparotomy, Jejunal repair, suture of the pancreatic head, distal pancreato-gastrostomy	ERCP—stenting of the ruptured pancreatic duct with 5 Ch pigtail drainage	ERCP unsuccessful, stenting of ruptured pancreatic duct not possible; chest drain (left side)
Postoperative Complications	Portal vein thrombosis with partial obstruction	None	Symptomatic pancreatic pseudocyst infection with *Clostridium difficile*	Symptomatic pancreatic pseudocyst *Candida albicans* sepsis; Posttraumatic stress disorder
Management of complications	Anticoagulant therapy	-	Transgastric punction and drainage of pseudocyst with double-pig tail drain Ch 7, spontaneous dislocation of pig tail Antibiotic therapy Persistent fistula of the ruptured pancreatic duct, recurrent stenting (2 times), removal of stent after 5 months	Transgastric punction and drainage of pseudocyst with double-pig tail Ch 7, spontaneous dislocation of pig tail Antifungal therapy
Duration of hospital stay	21 days	30 days	26 days	39 days
Follow Up	12 yrs	11.5 yrs	6.5 yrs	3.5 yrs

**Table 2:** Classification of pancreatic trauma according to the American Association for the Surgery of Trauma (AAST) [24].

Grading	Type of Injury	Description
**Grade I**	Hematoma Laceration	Minor contusion without duct injury Superficial laceration without duct injury
**Grade II**	Hematoma Laceration	Major contusion without duct injury or tissue loss Major laceration without duct injury or tissue loss
**Grade III**	Laceration	Distal transection or parenchymal injury with duct injury
**Grade IV**	Laceration	Proximal transection or parenchymal injury involving the ampulla
**Grade V**	Laceration	Massive disruption of the pancreatic head
